# Peculiar Properties of Template-Assisted Aniline Polymerization in a Buffer Solution Using Laccase and a Laccase–Mediator System as Compared with Chemical Polymerization

**DOI:** 10.3390/ijms241411374

**Published:** 2023-07-12

**Authors:** Olga Morozova, Irina Vasil’eva, Galina Shumakovich, Elena Zaitseva, Alexander Yaropolov

**Affiliations:** 1 A.N. Bach Institute of Biochemistry, Research Center of Biotechnology of the Russian Academy of Sciences, Leninsky Ave. 33, 119071 Moscow, Russia; morozova@inbi.ras.ru (O.M.); ir-vas@yandex.ru (I.V.); shumakovich1945@yandex.ru (G.S.); 2 Department of Chemistry, Lomonosov Moscow State University, Leninskie Gory 1/3, 119991 Moscow, Russia; ezaitseva2008@gmail.com

**Keywords:** enzymatic aniline polymerization, laccase, laccase–mediator system, chemical polymerization, oligoanilines, polyaniline

## Abstract

The conventional chemical polymerization of aniline has been described in multiple publications, while enzymatic polymerization has been poorly explored. A comparative study of the template-assisted enzymatic and chemical polymerization of aniline in a buffer solution of sodium dodecylbenzenesulfonate micelles was performed for the first time. The high-redox potential laccase from the fungus *Trametes hirsuta* was used as a catalyst and air oxygen served as an oxidant. Potentiometric and spectral methods have shown that oligomeric/polymeric products of the enzymatic polymerization of aniline are synthesized in the conducting emeraldine salt form immediately after the reaction is initiated by the enzyme. The use of the laccase–mediator system enabled a higher rate of enzymatic polymerization and a higher yield of final products. Potassium octocyanomolybdate (IV) served as a redox mediator. The products of the enzymatic polymerization of aniline were studied by the ATR-FTIR, MALDI-TOF and atomic force microscopy methods. The chemical oxidative polymerization of aniline under the same conditions resulted in forming a non-conducting dark brown product.

## 1. Introduction

**Figure 1:**
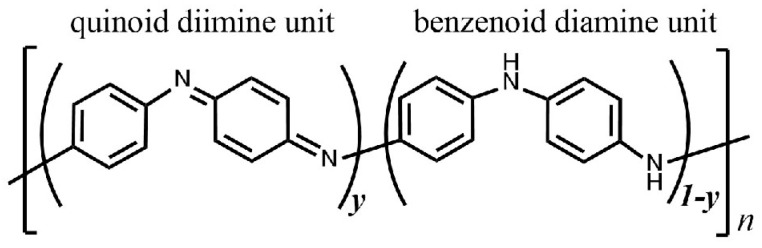
The general formula for polyaniline (PANI): *y* = 1, pernigraniline; *y* = 0, leucoemeraldine; *y* = 0.5, emeraldine.

Polyaniline (PANI) is one of the most extensively studied conducting polymers due to its stability under ambient conditions, easy synthesis, and the possibility to control its physicochemical properties by changing the oxidation state and the degree of protonation, allowing for its use in many areas [1,2,3,4,5]. PANI is known as a polymer that consists of oxidized and reduced repeat units [6]. The general formula of polyaniline is shown in Figure 1. There are three oxidation states of the polymer backbone: pernigraniline (the fully oxidized form), leucoemeraldine (the fully reduced form) and emeraldine (an intermediate oxidation state) [6]. PANI in the emeraldine oxidation state can participate in acid-based conversions and can exist both in the form of a conducting salt and a base.

Chemical oxidative polymerization is the oldest and yet the most popular method of PANI synthesis [7,8]. As a rule, ammonium peroxydisulfate is used as an oxidant. The chemical method of synthesis is far from being environmentally friendly, as it requires strong acidic media and large (monomer equivalent) amounts of the oxidant; it can also lead to the formation of toxic by-products such as benzidine. The chemically synthesized PANI has extremely low solubility in common solvents. Contrary to polyaniline, oligoanilines have a lower molecular mass, are soluble in many organic solvents and can be produced in a highly purified state. 

The mechanism of the conventional chemical polymerization of aniline in strong acidic media is not yet fully known [9,10,11,12]. It is believed that at the first stage of polymerization, unprotonated monomer molecules interact with the oxidant to form radical cations. Further anilinium cation radicals recombine to form the aniline dimer (N-phenyl-*p*-phenylenediamine). The formation of the aniline dimer is the limiting stage of polymerization that provides for an induction period in the reaction. The dimer and oligoanilines are characterized by lower redox potentials compared to the monomer. Thus, at subsequent stages of polymerization, the chain grows mainly through the oxidation of the terminal amino groups of oligomers to form active radicals that attack the monomer molecule at the para position [13]. Chemical polymerization proceeds via the stage at which aniline oligomers exist in the pernigraniline oxidation state. When the oxidant is consumed, the residial monomer slowly reduces pernigraniline to the emeraldine salt form of PANI. The mechanism of the chemical polymerization of aniline and the properties of the synthesized products are much dependent on the reaction conditions, including the pH value of the reaction medium, temperature and the monomer/oxidant ratio [8,13,14].

The enzymatic synthesis of PANI is an alternative to chemical polymerization and considerably meets the requirements of green and sustainable chemistry. Enzymatic reactions proceed under ‘mild’ operating conditions, namely, at pH values close to neutral, under atmospheric pressure, at room temperature, and in the absence of toxic organic solvents. Oxidoreductases (peroxidases and laccases) are mainly used as biocatalysts for the polymerization of arylamines [15,16,17,18,19,20,21,22]. Hydrogen peroxide is an oxidant in peroxidase-catalyzed reactions. However, peroxidases are highly sensitive to the hydrogen peroxide concentration and lose their activity at [H_2_O_2_] > 1 mM, which requires its gradual addition into the reaction medium. Some peroxidases, e.g., horseradish peroxidase, have a low stability at pH < 4 owing to their disassociation into the heme and apoenzyme. On the contrary, most laccases are acid stable, and air oxygen is an oxidant in laccase-catalyzed reactions.

Laccase (*p*-diphenol: oxygen oxidoreductase, EC 1.10.3.2) belongs to blue copper-containing oxidases that catalyze the one-electron oxidation of a broad range of compounds with the concomitant reduction of molecular oxygen to water. Substituted monophenols, polyphenols, aromatic amines and some inorganic compounds serve as laccase substrates [23,24]. The laccase-catalyzed oxidation of aromatic compounds proceeds via a radical mechanism with the subsequent combination of intermediates, which results in the formation of oligomeric and polymeric products [25,26]. The substrate specificity of laccases can be expanded using redox mediators, which are low molecular weight laccase substrates whose enzymatic oxidation leads to the formation of highly reactive intermediates [27]. These intermediates can oxidize the target substrate at a high rate. The use of laccase–mediator systems (LMS) in various areas is extensively covered in the literature [27,28,29,30,31].

In this work, the peculiar properties of the template-assisted polymerization of aniline using laccase and a laccase–mediator system in a buffer solution were studied in comparison with chemical polymerization under the same conditions.

## 2. Results and Discussion

The polymerization of aniline was studied in a 0.1 M Na-citrate-phosphate-buffered solution (pH 3.7). Since oligoanilines and PANI are virtually insoluble in aqueous solutions, the use of templates in aniline polymerization enables polymer/template colloidal dispersions to be obtained, which simplifies the study of the reaction in the bulk solution. The surfactant sodium dodecylbenzenesulfonate (DBSNa) was used as both a template and a dopant of the polymer backbone. DBSNa is able to form stable micellar structures in aqueous solutions if its concentration is higher than the critical micellar concentration (CMC_DBSNa_ = 1.6 mM). The fungal laccase *Trametes hirsuta* was used as a biocatalyst in the oxidative polymerization of aniline. The redox potential of the primary electron acceptor of the enzyme (T1 center) was 0.78 V vs. NHE [32]. Atmospheric oxygen served as the terminal oxidant.

The addition of aniline to a 20 mM micellar solution of DBSNa in the buffer produced a white turbid dispersion of aniline/DBSNa particles. A similar effect was described in [33], where the dodecylbenzenesulfonic acid was used as a surfactant for chemical aniline polymerization. A photograph of the resulting aniline/DBSNa complexes is shown in Supplementary Figure S1. Our preliminary experiments demonstrated that the aniline/DBSNa complexes prevented the denaturation of laccase by the surfactant. The template-assisted aniline polymerization was monitored by UV–vis spectroscopy and by measuring the open-circuit potential of the reaction mixture.

### 2.1. Laccase-Catalyzed Aniline Polymerization

**Figure 2:**
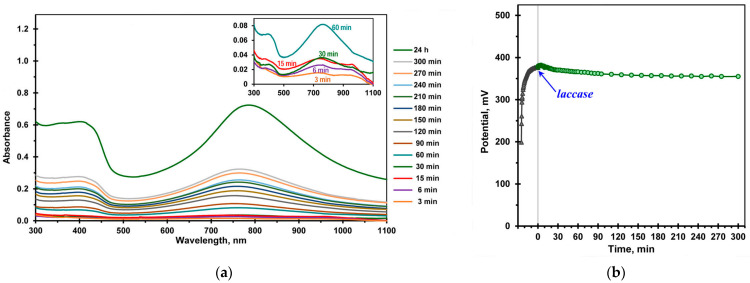
Laccase-catalyzed template-assisted aniline polymerization in a buffer solution. (**a**) The evolution of the UV–vis absorbtion spectra. The samples were diluted 40 times with a 20 mM DBSNa buffer solution. Insert: early stages of the reaction; (**b**) potential–time profile before (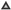) and after (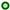) adding laccase.

Figure 2 shows the evolution of UV–vis absorption spectra and the change in the potential of the reaction medium during the laccase-catalyzed aniline polymerization. After the reaction was initiated by the enzyme, the color of the reaction mixture quickly changed from pale turquoise (3 min) to sea green (15 min) and finally to dark green (24 h) (Supplementary Figure S2), which indicates that oligoanilines were synthesized in the emeraldine salt form from the first minutes of the reaction. It should be noted that the pH value of the reaction medium did not change during 24 h of the laccase-catalyzed reaction.

As can be seen in Figure 2a, an absorption maximum around 760 nm and a flat in the region of 320–420 nm appeared in the third minute of the enzymatic reaction. It is known that the spectra of doped PANI show three typical absorption bands at 325–360, 400–430 and 780–826 nm [33,34]. The first two bands are often combined into a flat or distorted single peak with a local maximum between 380 and 420 nm. The absorption near 780–826 nm is attributed to the presence of a polaron in the PANI structure [34]. During the laccase-catalyzed aniline polymerization, the absorption in these regions increased (Figure 2a), and the maximum corresponding to the polaron shifted to the long-wavelength region of the spectrum from 760 nm (3–15 min of the reaction) to 787 nm (24 h).

During the laccase-catalyzed aniline polymerization, the redox potential of the reaction medium gradually decreased from the initial value of 377 mV (immediately after the addition of the enzyme) to a value of 359 mV after 120 min (Figure 2b). Apparently, the potential decrease is due to the fact that oligoanilines, which have lower redox potentials compared to the monomer, begin to form from the first minutes of the reaction [11]. The data of UV–vis spectroscopy enable us to assume that these oligoanilines are in the emeraldine salt form.

### 2.2. Aniline Polymerization Using a Laccase–Mediator System

The rate of the laccase-catalyzed polymerization of aniline is relatively low due to the high oxidation potential of the monomer. An LMS based on potassium octocyanomolybdate (IV) K_4_Mo(CN)_8_ (E^0^ = 0.78 V *vs*. NHE) was used to accelerate the enzymatic reaction. It was shown in preliminary experiments that *T. hirsuta* laccase catalyzes the oxidation of K_4_Mo(CN)_8_ at acidic pH values (3.0–4.5) with the catalytic efficiency k_cat_/K_m_ = 0.26 μM^−1^ s^−1^, which enables its use as a redox mediator.

After the reaction was initiated by the enzyme, the color of the reaction mixture quickly changed from white to sea green (3 min) and finally to dark green (24 h) (Supplementary Figure S3). The pH value of the reaction medium did not change during 24 h, as was the case with the laccase-catalyzed polymerization of aniline.

**Figure 3:**
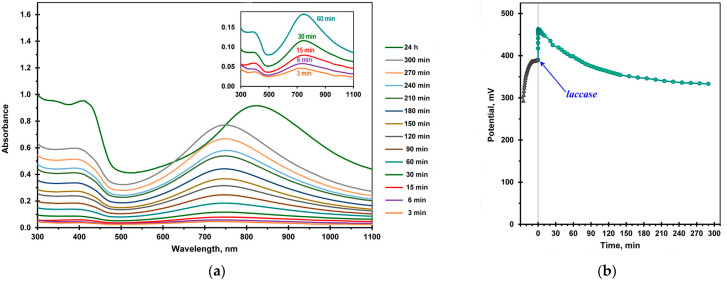
Aniline polymerization using a laccase–mediator system. (**a**) The evolution of the UV–vis absorbtion spectra. The samples were diluted 40 times with a 20 mM DBSNa buffer solution. Insert: Early stages of the reaction; (**b**) potential–time profile before (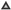) and after (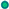) adding laccase.

Figure 3 shows the UV–vis absorption spectra and the change in the potential of the reaction medium during the oxidative aniline polymerization using LMS. The shapes of the spectra (Figure 3a) are similar to those of the oligoanilines produced in the laccase-catalyzed reaction (Figure 2a). However, the polaron absorption maximum is shifted to the long wavelength part of the spectrum up to 825 nm after 24 h of polymerization, which can be associated with both a higher polymerization degree of aniline and a different ratio of oxidized and reduced units in the polymer backbone. The use of the redox mediator enabled an approximately five-fold increase in the rate of the enzymatic polymerization of aniline.

After the reaction was initiated by the enzyme, the potential of the reaction medium drastically increased from the initial value of 390 mV to 463 mV (Figure 3b), which was associated with the rapid enzymatic oxidation of K_4_Mo(CN)_8_. It was shown in an independent experiment that the same increase in the potential occurred when laccase was added to a buffer solution containing K_4_Mo(CN)_8_ (without aniline and SDBSNa). Afterwards, as was the case with the laccase-catalyzed synthesis, the potential of the reaction medium gradually decreased. However, the rate of the potential decrease was several times higher, which is probably due to the involvement of the oxidized form of the redox mediator in the nonenzymatic oxidation of both the monomer and oligoanilines.

**Figure 4:**
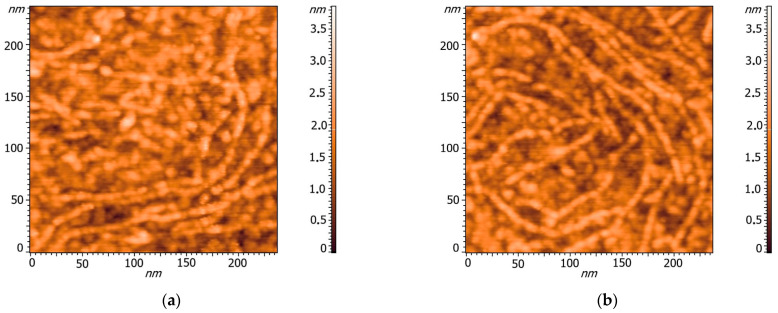
AFM images of PANI/DBSNa complexes synthesized using (**a**) laccase and (**b**) LMS.

It should be noted that when the mediator was used, the yield of the end products of aniline polymerization increased from 28.6 ± 0.4 to 49.3 ± 0.3% after 24 h. Besides, the conductivity values of the PANI/DBSNa complexes synthesized using laccase and LMS were in the ranges of 0.3–0.5 mS cm^−1^ and 1–2 mS cm^−1^, respectively. An AFM study of the PANI/DBSNa complexes showed a morphological similarity between both enzymatically synthesized samples (Figure 4). The complexes had a granular form and combined into agglomerates.

**Figure 5:**
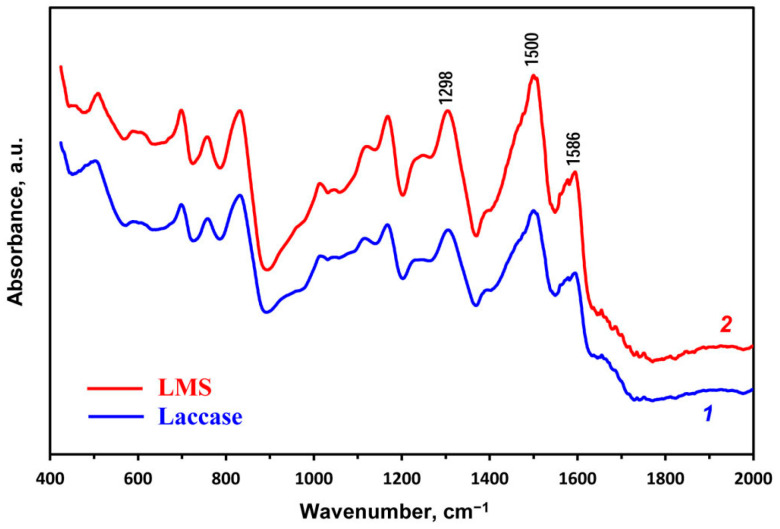
ATR-FTIR spectra of dedoped aniline polymerization products synthesized by the laccase-catalyzed method (1) and with the use of LMS (2).

The ATR-FTIR spectra of dedoped PANI synthesized using laccase and LMS are quite similar (Figure 5). Both spectra have characteristic peaks at 1500 and 1586 cm^−1^ assigned to the C–C aromatic ring stretching of the benzenoide diamine units and both C=N and C=C stretching of the quinoid diimine units, respectively. The band at 1298 cm^−1^ is due to a mixed mode of the C–H bending and the C–N stretching vibrations [35]. The peak intensity ratio of the quinoid diimine/benzenoide diamine units indicates the degree of PANI oxidation. This ratio is ~0.8 for the emeraldine base, and it decreases as the PANI backbone is reduced [35]. For PANI synthesized using laccase and LMS, this ratio was 0.73 and 0.75, respectively. The bands at 1000–1250 cm^−1^ are due to the DBSNa template. Apparently, some of the template molecules remain firmly bound to the PANI backbone despite the hard dedoping conditions.

**Figure 6:**
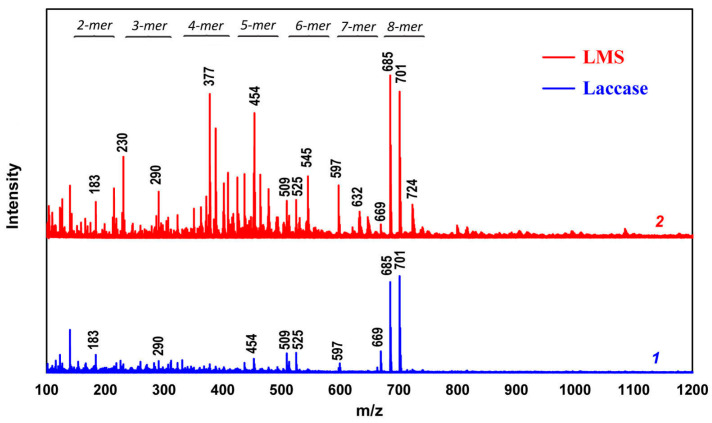
MALDI-TOF spectra of dedoped oligoanilines synthesized using laccase (1) and LMS (2).

The products of aniline polymerization on the SDBNa template obtained using laccase and LMS after a four-hour reaction were analyzed by the MALDI TOF method (Figure 6). Prior to mass spectral analysis, the products were dedoped with an aqueous ammonia solution, the precipitate was washed first with an ethanol/water mixture (50/50 vol.%) and then repeatedly with deionized water, and dried. The resulting samples were completely soluble in tetrahydrofuran. The mass spectra of both samples had the peaks that can be attributed to 2–8 mers. The peaks corresponding to the 8 mers are dominant in both spectra. It should be noted that all the peaks are grouped into multiplets, which is associated with different end groups and the oxidation degree of the synthesized oligoanilines [19,36,37,38,39]. However, the number of different redox states observed for n-mers synthesized using LMS is significantly higher than when laccase is used alone.

### 2.3. Chemical Aniline Polymerization Using Ammonium Peroxydisulfate as an Oxidant in a Buffer Micellar Solution

**Figure 7:**
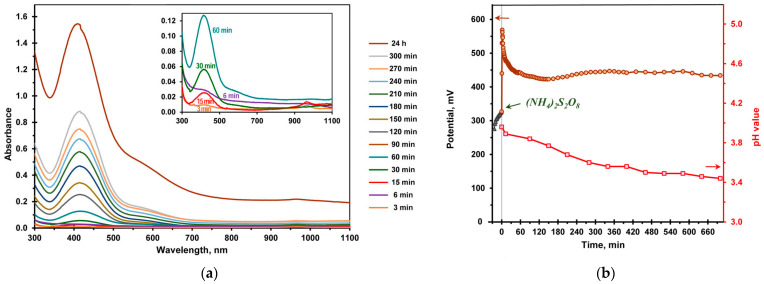
Chemical aniline polymerization with ammonium peroxydisulfate as an oxidant in a DBSNa micellar solution. (**a**) The evolution of the UV–vis absorbtion spectra. The samples were diluted 20 times with a 20 mM DBSNa buffer solution. The spectrum after 24 h of the reaction recorded at 40-fold dilution. Insert: early stages of the reaction; (**b**) the plot of the potential before (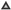) and after (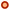) adding (NH_4_)_2_S_2_O_8_ and pH (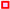) of the reaction media as functions of time.

The process of the chemical-template-assisted polymerization of aniline in a buffer solution differs from that of enzymatic polymerization. The evolution of UV–vis absorption spectra and the change in the potential and pH of the reaction medium during the chemical polymerization of aniline are shown in Figure 7.

An absorption band with a maximum at 415 nm appeared in the spectrum 15 min after the initiation of the reaction with ammonium peroxydisulfate (Figure 7a). The absorbtion at this wavelength increased with time and the position of the maximum did not change. The micellar solution turned pale yellow in the 6th min of the reaction and 6 h later it became dark brown (Supplementary Figure S4). In the chemical aniline polymerization, the pH value of the medium dropped to pH 3.44 by the 700th min, in contrast to the laccase-catalyzed and LMS syntheses (Figure 7b). It is noteworthy that the end product of the reaction was dark brown and non-conducting.

The potential of the reaction medium drastically increased by approximately 240 mV immediately after the addition of (NH_4_)_2_S_2_O_8_ (Figure 7b). Then, the potential gradually decreased by 130 mV within 60 min and afterwards remained unchanged. The data obtained correlate with the results presented in [14]. Surwade et al. studied the template-free chemical polymerization of aniline in buffer solutions and proposed a new mechanism for the formation of oligoanilines via the intermediacy of benzoquinone monoimine.

Thus, as a result of template-assisted aniline polymerization in the buffer solution using laccase and LMS, we obtained conducting polyaniline in the emeraldine salt form, while the products of the chemical aniline polymerization were non-conducting (Table 1). 

## 3. Materials and Methods

### 3.1. Materials

Aniline (Sigma-Aldrich, Saint Louis, MO, USA) was distilled in vacuo before use. Na_2_HPO_4_‧2H_2_O (Riedel-de Haën, Seelze, Germany), 2,2-azino-bis(3-ethyl-benzthiazoline-6-sulfonate (ABTS), citric acid anhydrous, sodium dodecylbenzenesulfonate (DBSNa), potassium octacyanomolybdate (IV) hydrate (Sigma-Aldrich, Steinheim, Germany), ammonium peroxodisulfate (Carl Roth GmbH, Karlsruhe, Germany), and tetrahydrofurane (Lab-Scan, Gliwice, Poland) were used without further purification. All the solutions were prepared using water purified with a Simplicity^®^ Water Purification System (Merck KGaA, Darmstadt, Germany).

### 3.2. Enzyme

A laccase from the fungus *Trametes hirsuta* (Wulfen) Pilát CF-28 was purified to homogeneity, as described in [40]. The specific activity of the enzyme stock solution was about 138 U mg^−1^ of protein. The protein concentration was *ca.* 7.4 mg mL^−1^. The laccase activity, using ABTS as a chromogenic substrate, and protein concentration were measured spectrophotometrically as described previously [41].

### 3.3. Template-Assisted Aniline Polymerization in a Buffer Solution

The reaction was carried out in a 0.1 M Na-citrate-phosphate buffer (pH 3.7). The surfactant DBSNa was used as a template. In order to prepare the aniline/DBSNa complex, 69.8 mg of DBSNa was dissolved in 10 mL of the buffer. Then, 18.3 μL of aniline was added dropwise and the solution was stirred for 1 h until a white turbid dispersion of aniline/DBSNa particles was obtained. The concentration of DBSNa and aniline in the reaction mixture was 20 mM.

Laccase-catalyzed aniline polymerization was initiated by adding a laccase stock solution. The specific activity of the enzyme in the reaction mixture was ~1.0 U mL^−1^. The reaction was performed at room temperature (21–22 °C) under aerobic conditions and with constant stirring on a magnetic stirrer for 24 h. When the laccase mediator system was used, 0.05 mL of a 10 mM solution of the K_4_Mo(CN)_8_ mediator in a buffer (mediator concentration in the reaction medium 0.05 mM) was added to the reaction mixture before initiating the reaction with the enzyme.

The chemical polymerization of aniline was performed under the same conditions with minor changes: DBSNa and aniline were dissolved in 9.5 mL of the buffer. The reaction was initiated with 0.5 mL of a 0.4 M solution of ammonium peroxodisulfate (APS) added dropwise in the same buffer. The concentration of DBSNa, aniline and APS in the reaction mixture was 20 mM.

The template-assisted aniline polymerization was monitored by UV–vis spectroscopy using a Shimadzu UV1240 mini spectrophotometer (SHIMADZU CORPORATION, Kyoto, Japan) and by measuring the open-circuit potential of the reaction mixture using a BAS CV-50W voltammetric analyzer (Bioanalytical Systems Inc., West Lafayette, IN, USA) and a single-compartment two-electrode cell. A gold wire and Ag/AgCl electrode (BAS) served as working and reference electrodes, respectively.

In order to produce dedoped oligo-/polyanilines, the resulting products were treated with 3% aqueous ammonia for 24 h and then diluted with an equal volume of ethanol. The precipitate was separated by centrifugation, washed with deionized water, and dried for 48 h at 70 °C.

To measure the conductivity of the end products, a double amount of ethanol was added into the PANI/SDBS dispersion to destroy the micelles and precipitate PANI. The precipitate was separated by centrifugation, washed with ethanol/water mixture (50/50 vol.%) and dried for 48 h at 70 °C.

### 3.4. Characterization of the Products

ATR-FTIR-spectra were recorded on a Spectrum Two™ FT-IR spectrometer (PerkinElmer Inc., Waltham, MA, USA). The MALDI-TOF spectra of dedoped oligoanilines were recorded in the reflection mode using a Brucker Daltonocs Micriflex mass spectrometer (Bruker Daltonics GmbH & Co. KG, Bremen, Germany), which was calibrated with peptides with known molecular masses from 700 to 3500 Da. The morphology of the products was studied using a SmartSPM 1000 Scanning Probe Microscope (AIST-NT, Moscow, Russia) on the surface of highly oriented pyrolytic graphite (Advanced Technologies Centre, Russia). Optical imaging of the API/DBS complex was obtained using an Olympus BX41 Microscope (Olympus Corporation, Tokyo, Japan). Four-point conductivity measurements were carried out with a Loresta GP MCP-T610 resistivity meter (Mitsubishi Chemical Analytech Co., Chigasaki, Kanaga-wa, Japan) using an MCP-TP06P probe (inter-pin distance 1.5 mm, pin points 0.26 R, spring pressure 70 g pin^−1^).

## 4. Conclusions

It has been shown that in the aniline polymerization in a micellar buffer solution using laccase and a laccase–mediator system, oligoanilines were synthesized in the emeraldine salt form immediately after the initiation of the reaction by the enzyme. In both cases, conducting products containing benzenoid diamine and quinoid diimine units were formed. The enzymatic polymerization of the monomer was greatly accelerated, and the yield of the resulting products was increased through the use of the redox mediator. It should be noted that the enzymatic reaction can be stopped at any time by increasing the pH of the reaction medium up to pH > 7.5. As compared to the enzymatic polymerization, the end product of the chemical aniline polymerization performed under the same conditions was dark brown and non-conducting.

## Figures and Tables

**Table 1:** Conditions of template-assisted aniline polymerization in a buffer solution * and characteristics of the end products.

Synthesis	Oxidant	Polymerization Time, h	UV–Vis Absorption Characteristics, nm	Conductivity, mS cm^−1^
Laccase-catalyzed	Air oxygen	24	380–420, 787	0.3–0.5
LMS (laccase + K_4_Mo(CN)_8_)	Air oxygen	24	380–420, 825	1–2
Chemical	(NH_4_)_2_S_2_O_8_	24	415	non-conducting

* 0.1 M Na-citrate-phosphate buffer (pH 3.7), template—sodium dodecylbenzenesulfonate (DBSNa), [DBSNa] = [aniline] = 20mM, room temperature (21–22 °C).

## Data Availability

Not applicable.

## Supplementary Materials

The following supporting information can be downloaded at: https://www.mdpi.com/article/10.3390/ijms241411374/s1.
