# Supplementary material for: Peculiar Properties of Template-Assisted Aniline Polymerization in a Buffer Solution Using Laccase and a Laccase–Mediator System as Compared with Chemical Polymerization
Source: Int J Mol Sci. 2023 Jul 12;24(14):11374. doi: 10.3390/ijms241411374 (PMC10380230; doi:10.3390/ijms241411374)
Supplement: Supplementary file 1 [file ijms-24-11374-s001.zip › ijms-2496473-supplementary.pdf]

# Supplementary Materials

# Peculiar Properties of Template-Assisted Aniline Polymerization in a Buffer Solution Using Laccase and a Laccase–Mediator System as Compared with Chemical Polymerization

**Olga Morozova [1], Irina Vasil’eva [1], Galina Shumakovich [1], Elena Zaitseva [2] and Alexander Yaropolov [1,*]**

1 A.N. Bach Institute of Biochemistry, Research Center of Biotechnology of the Russian Academy of Sciences, Leninsky Ave. 33, 119071 Moscow, Russia
2 Department of Chemistry, Lomonosov Moscow State University, Leninskie Gory 1/3, 119991 Moscow, Russia
* Correspondence: yaropolov@inbi.ras.ru or alexander-yaropolov52@yandex.ru

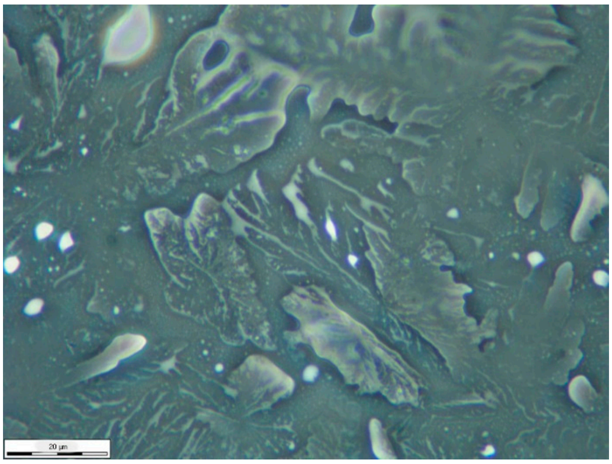


**Figure S1.** An optical image of aniline/DBSNa complexes.

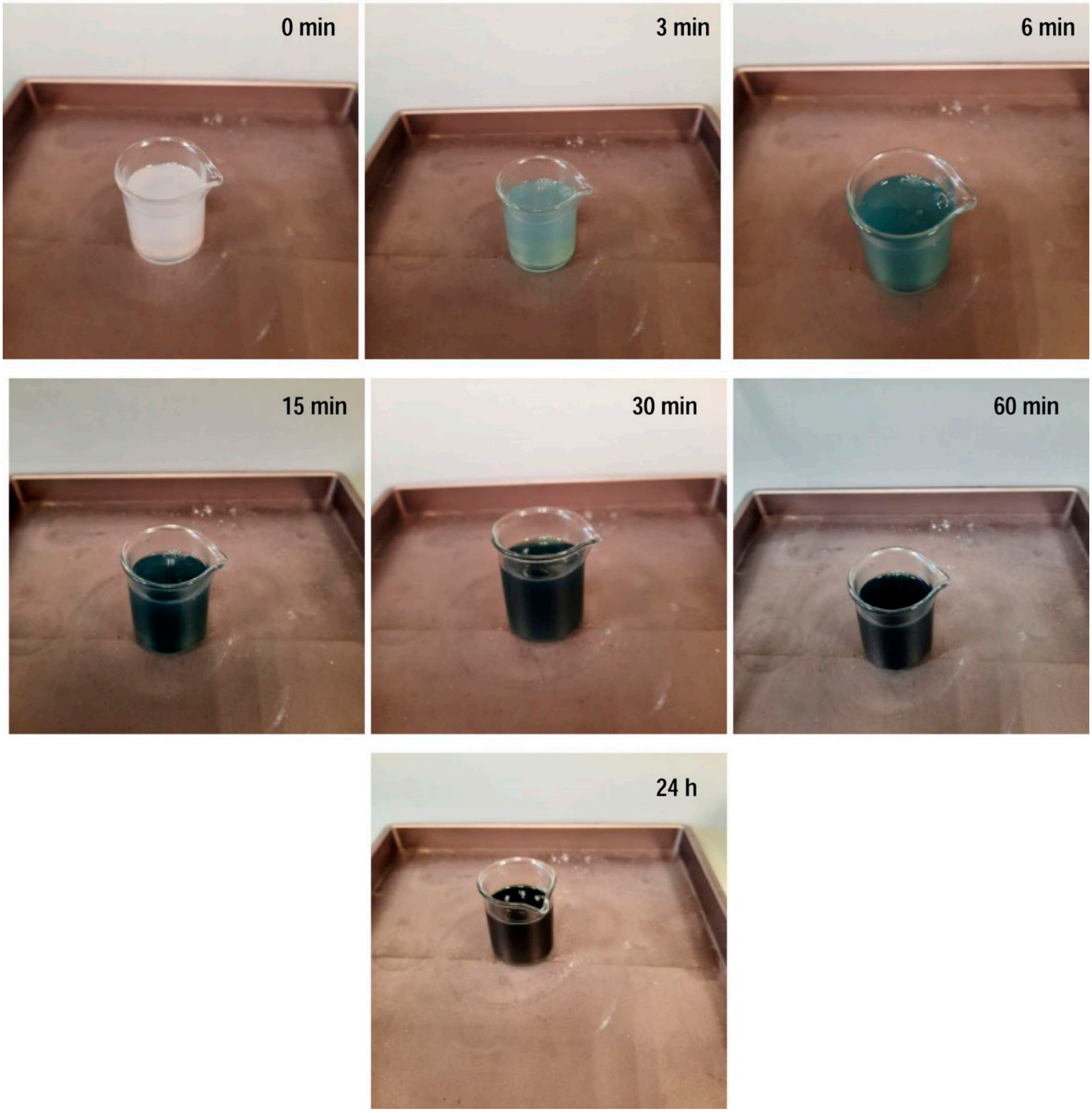


**Figure S2**. Photographs of the reaction medium during the laccase-catalyzed template assisted aniline polymerization in a buffer solution.

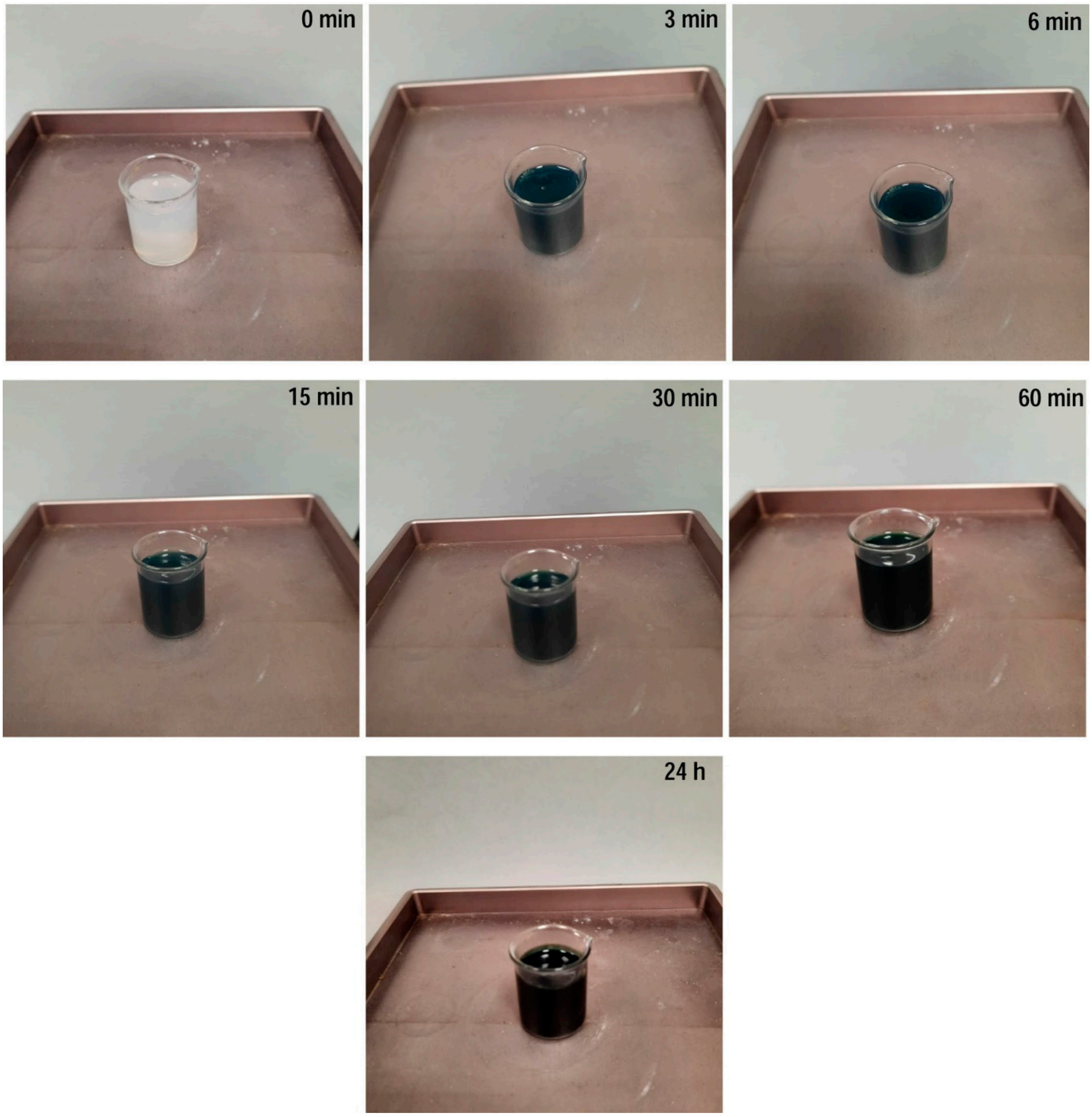


**Figure S3**. Photographs of the reaction medium during the template assisted oxidative polymerization of aniline in a buffer solution using LMS.

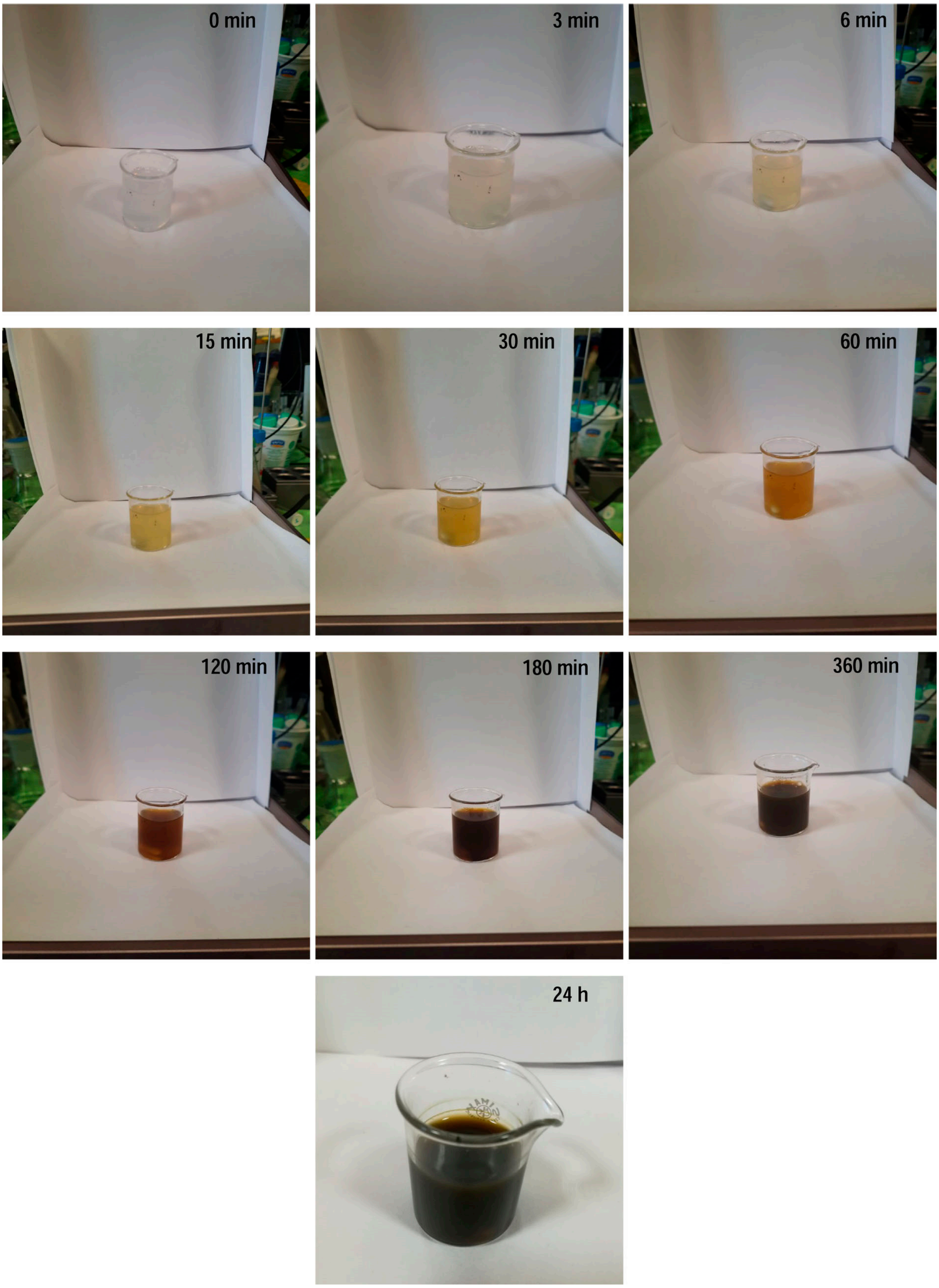


**Figure S4**. Photographs of the reaction medium during the chemical aniline polymerization using ammonium peroxydisulfate as an oxidant in a buffer micellar solution.
